# Effects of intranasal oxytocin administration on empathy and approach motivation in women with borderline personality disorder: a randomized controlled trial

**DOI:** 10.1038/s41398-019-0658-4

**Published:** 2019-12-04

**Authors:** Gregor Domes, Nicole Ower, Bernadette von Dawans, Franny B. Spengler, Isabel Dziobek, Martin Bohus, Swantje Matthies, Alexandra Philipsen, Markus Heinrichs

**Affiliations:** 1 Department of Psychology, Laboratory for Biological and Personality Psychology, University of Freiburg, Freiburg, Germany; 2 Department of Biological and Clinical Psychology, University of Trier, Trier, Germany; 3 Department of Psychology, Clinical Psychology of Social Interaction, Humboldt-Universität zu Berlin, Berlin, Germany; 4 Institute of Psychosomatic and Psychiatric Psychotherapy, Central Institute of Mental Health, Heidelberg University, Mannheim, Germany; 5 McLean Hospital Harvard Medical School, Boston, MA USA; 6 Department of Psychiatry and Psychotherapy, Medical Center - University of Freiburg, Faculty of Medicine, University of Freiburg, Germany; 7 Department of Psychiatry and Psychotherapy, University Hospital Bonn, Bonn, Germany; 8 Freiburg Brain Imaging Center, University Medical Center, University of Freiburg, Freiburg, Germany

**Keywords:** Human behaviour, Psychiatric disorders

## Abstract

Borderline personality disorder (BPD) is characterized by severe interpersonal dysfunction with problems in social cognition, empathy and social approach. Although the neuropeptide oxytocin is known to regulate complex social cognition and behavior in healthy individuals and clinical populations, there is still a lack of evidence for a potential beneficial effect of oxytocin administration on social cognition and social approach in BPD. Fifty-one women with BPD and 51 matched healthy controls were randomized to a double-blind, placebo-controlled, between-subject experimental trial. We administered a single dose of 24 IU oxytocin or placebo intranasally prior to a standardized task measuring affective and cognitive empathy and approach motivation. All participants were free of hormonal contraception and tested in the mid-luteal phase of their menstrual cycle. In the placebo condition, patients with BPD showed reduced cognitive and affective empathy, and less approach behavior motivation than healthy controls. Intranasal oxytocin significantly increased affective empathy and approach motivation in both BPD patients and healthy controls compared to placebo. More importantly, oxytocin administration led to similar scores between BPD and healthy controls. These findings provide the first evidence for a beneficial effect of oxytocin on deficits in affective empathy and approach motivation of BPD. Our results indicate a beneficial effect of a single dose of oxytocin on affective empathy and approach motivation in women with BPD adapting their level of social functioning to healthy controls. Future clinical trials will need to investigate the long-term effects and effectiveness of oxytocin as an add-on treatment for social impairments in BPD.

## Introduction

Borderline personality disorder (BPD) is a severe psychiatric disorder characterized by a pervasive pattern of instability in relationships, affect and self-image. With a lifetime prevalence of about 2%, BPD patients account for 10% of all psychiatric outpatients and for about 20% of inpatients and thus cause tremendous costs to health systems^1^. To date, there is no specific pharmacological treatment available, and psychotherapy only reveals effects in parts of the patient population^1^. BPD patients often report problems establishing and maintaining stable relationships to significant others^2^. Their relationships are characterized by a pervasive fear of abandonment, anxiousness, mistrust, and conflicts which can culminate in hostile and impulsive behavior^3^. Interpersonal problems are among the core symptoms of the disorder, and difficulties in social interactions and social relationships are associated with disadvantages in broader social functioning and social integration^4,5^.

Empathy as a prerequisite for intact social functioning comprises both the cognitive ability to recognize others’ emotional states, and the affective process of vicariously feeling others’ emotions^6^. Both facets of empathy form the basis for understanding and predicting other people’s behavior and for regulating one’s own reactions in social interactions. Although impairments in empathy have been discussed as a psychopathological factor in BPD and are defined as a core feature of BPD in the DSM-5^3^, the empirical evidence for impaired empathy in BPD is controversial^7,8^. Some studies have reported enhanced affective responding to social stimuli and better emotion recognition^9–11^, while others using more complex tasks demanding higher order integration of social information, resembling the challenges of real-life social situations, demonstrate empathic impairments in BPD^12–14^.

Oxytocin (OT) is a neuropeptide that is essentially involved in social cognition, behavior, and brain structure^15–17^. In humans, OT exerts behavioral and neural effects associated with social approach and avoidance: it reduces anxiety and the endocrine responses to social stressors^18,19^, enhances trust in social interactions^20,21^ and strengthens positive communication in couples^22^. In addition, a number of studies provided evidence for enhanced emotion recognition^23–25^ and empathy^26,27^ following OT administration.

There is initial evidence for lower peripheral OT-levels in women with BPD, suggesting altered endogenous OT-signaling in these patients^28^. The exogenous administration of OT has been reported to reduce stress reactivity in BPD^29^, reduce avoidant reactions to threat-signaling social stimuli^30^ and attenuate the emotional and neural responses to threat-related social signals^31^ and emotional stimuli in general^32^. Taken together, these studies suggest altered endogenous OT-signaling in women with BPD, and positive effects of exogenous OT on stress reactivity as well as emotional and neural responses to threatening social stimuli^5^, although others have reported impaired trusting behavior after OT administration^33,34^.

Based on these findings, the present study aimed to investigate OT’s effects on different facets of empathy and approach motivation in BPD as compared to healthy controls by assessing stimulus-based responses. Using a double-blind, placebo-controlled, between-subject design, we hypothesized that a single dose of OT given intranasally would enhance both cognitive and emotional empathy and approach motivation in BPD and a healthy control group. Including a healthy control group further allowed us to clarify whether oxytocin would normalize decreased baseline empathy and approach motivation in BPD patients to the level of healthy subjects.

## Materials and methods

### Participants

**Fig. 1:**
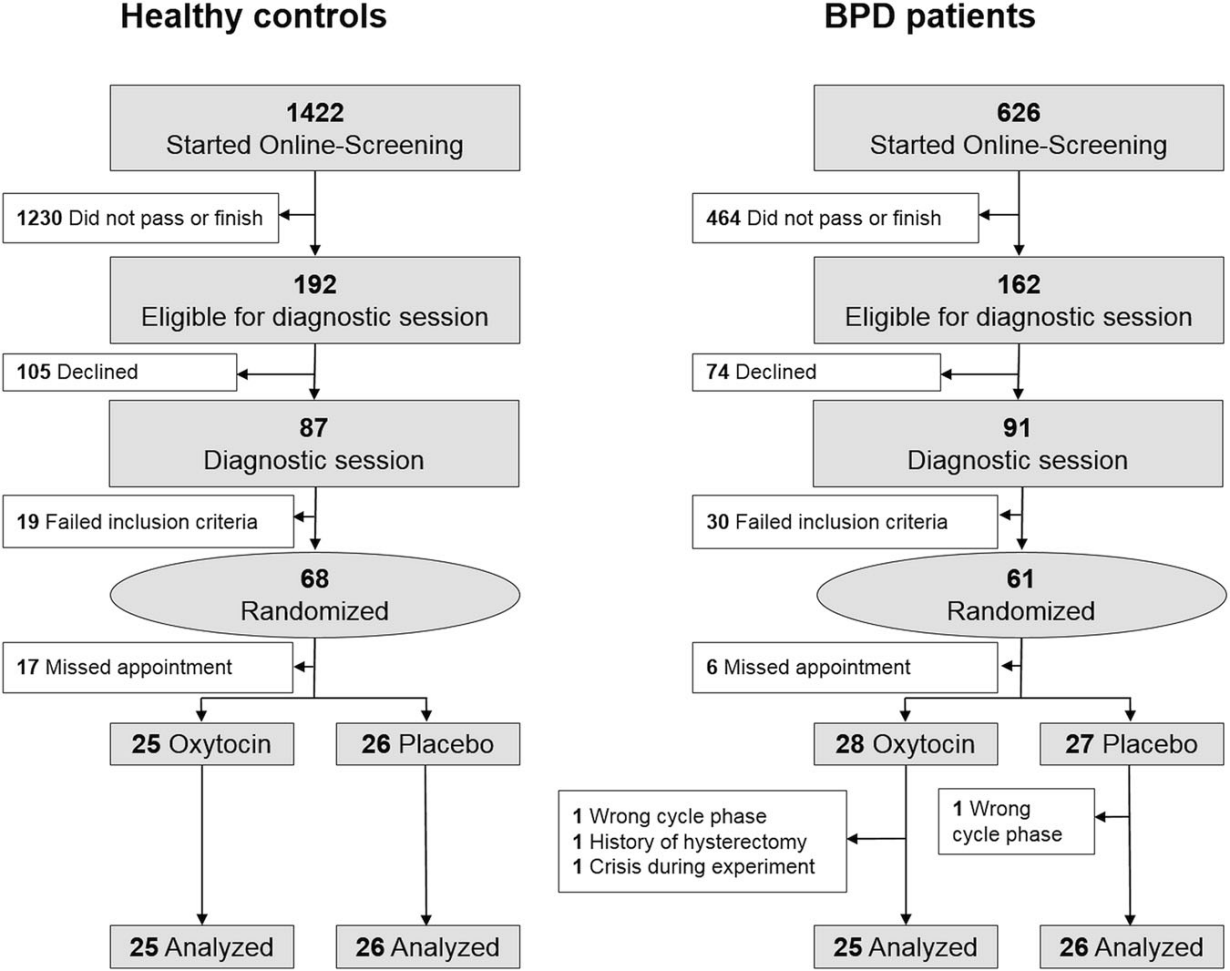
Enrollment and randomization of participants.

In total, 61 women diagnosed with BPD and 68 healthy women, matched for age and education, were enrolled for the study (details on sample size calculation are found in the supplemental methods). Axis I and II psychiatric disorders were diagnosed via the Mini-International Neuropsychiatric Interview^35^ and the Structured Clinical Interview for DSM-IV Axis II Disorders^36^. To assess severity of BPD symptomatology patients underwent the Rating Scale for Borderline Personality Disorder (ZAN-BPD)^37^ and the Borderline Symptom List (BSL)^38^. For the description of the study groups, all participants completed a set of questionnaires regarding demographic and psychopathological characteristics (for details see supplemental methods). Data of 51 BPD patients and 51 healthy controls were available for final analyses (see Fig. 1 for detailed description of enrollment and drop-outs).

All participants were free of hormonal contraception for at least 3 months and were tested in their mid-luteal cycle phase (day 18 to day 25 of the ovarian cycle) as assessed by self-report. Healthy controls were excluded from participation if they reported any Axis I and II psychiatric disorder. BPD patients were excluded if they met criteria for lifetime bipolar disorder, lifetime schizophrenia, and current alcohol or substance addiction. In both groups, pregnant and breast-feeding women were also excluded. All participants provided written-informed consent for the experimental procedures. The study was approved by the ethics committee of the University of Freiburg and registered as a clinical trial at clinicaltrials.gov (NCT01243658).

### Measures

**Fig. 2:**
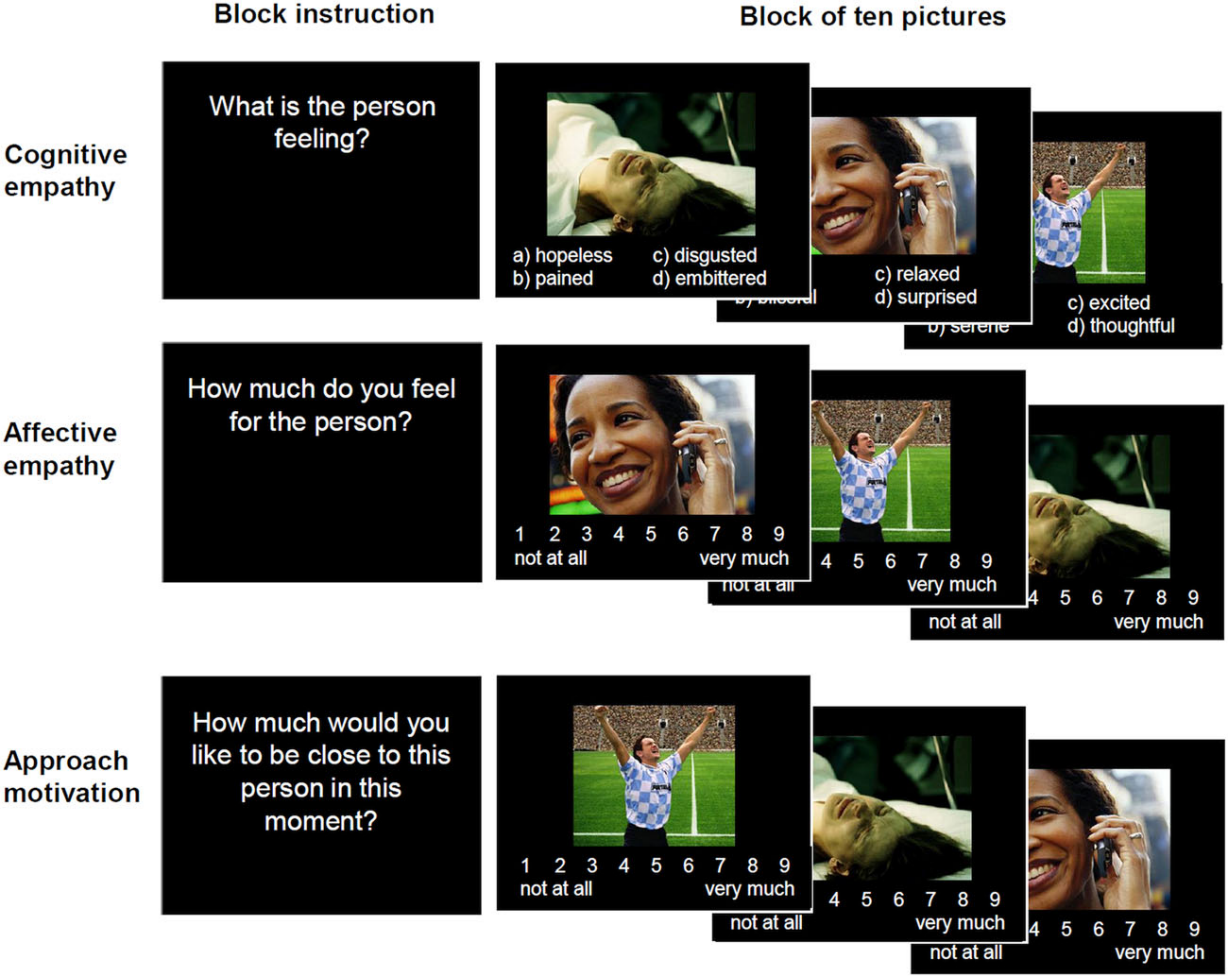
Task design of the MET. Stimuli were randomized and presented in blocks of ten pictures. Each block was introduced by a specific instruction.

To assess empathy and approach motivation, we applied an extended version of the Multifaceted Empathy Test, MET^39^. The MET is an ecologically valid instrument to assess cognitive and emotional facets of empathy. The MET version used in this and previous studies^27,40^ consists of 30 pictures showing people in emotionally-laden situations. In this version, 13 of the pictures depict people experiencing positive emotions (e.g., a sportsman being proud about winning a game or a child feeling happy cuddling its pet), whereas seventeen of the pictures show people experiencing negative emotions (e.g., a women feeling depressed after having been slapped or a crying child in a war scene). To assess cognitive empathy, participants were asked to select the correct mental state description out of four labels given below the picture. To measure affective empathy, participants are asked to rate in separate blocks their experienced level of empathic concern for the person in the picture on a 9-point Likert scale. Approach motivation was assessed by asking the participants to rate their desire to be close to the depicted person on another 9-point Likert scale. For each measure (cognitive and emotional empathy, and approach motivation), stimuli were presented in blocks of ten pictures, each picture followed by the respective question. Thus, all pictures were presented three times with the specific instruction for empathy and approach motivation assessment. For each participant, pictures were randomly assigned to the three blocks and presented in random order (see Fig. 2). For the whole group of participants, the linear association between affective empathy and approach motivation was high (r = .83, *p* < .001), between affective and cognitive empathy modest (r = .33; *p* < .001), and for cognitive empathy and approach motivation non-significant (r = .18; *p* = .06).

Trial presentation and response registration was controlled by a PC running Presentation (Neurobehavioral Systems Inc, Albany, CA, USA). For statistical analyses, we calculated total scores for each task and subscores for positive and negative stimuli, by averaging the trials within a specific condition. To control for non-specific substance effects, we collected the following state measures: mood, using the Multimodal Mood Questionnaire (MDBF), anxiety using the State-Trait-Anxiety-Inventory (STAI), anger using the State-Trait-Anger-Inventory (STAXI) and distress with a visual analog scale (VAS) at three times during the time-course of the experiment: before substance application (t_1_), shortly before the experiment (t_2_) and directly after the experiment (t_3_).

### Treatment and procedures

Participants were randomly assigned to receive OT or placebo in a double-blind between-subject study design. Forty-five minutes before the empathy task started, participants self-administered 24 IU OT (or the placebo) via nasal spray (Syntocinon nasal spray, three puffs per nostril, with 4 IU per puff) under the experimenter’s supervision to ensure standardized substance uptake. The placebo contained exactly the same ingredients as the OT spray except for the active agent to avoid subjective substance effects (e.g., olfactory effects). Nasal sprays were blinded by the pharmacy.

### Analysis

Group and drug effects on empathy and approach motivation were tested with separate 2-way univariate analyses of variance for total scores, and positive and negative stimuli. Two-way ANOVAs and three-way ANOVAs with time as a repeated measure were calculated to test for differences and changes in mood, anxiety, anger, and tension over the course of the experiment. To control for possible moderating effects of symptom severity within the patient group, ANCOVAs were conducted with the total scores of the Borderline Symptom List (BSL)^38^ and the Childhood Trauma Questionnaire (CTQ). Levene’s test was used to assess the equality of variances. For all analyses, the level of significance was set to *p* *<* 0.05 (two-tailed). Effect sizes are reported as partial η^2^ and Cohen’s d. All analyses were calculated with SPSS, version 25 (SPSS, Chicago).

## Results

### Sample characteristics

BPD patients and healthy controls did not differ in age, education and verbal IQ (vocabulary test). With regard to all psychopathological variabIes (see a detailed description see supplementary materials), BPD patients exhibited significantly higher scores compared to the healthy control group (see Table 1).

**Table 1:** Demographic and psychopathological characteristics (mean values and standard deviation in parantheses) of healthy controls and patients with Borderline personality disorder.

	Healthy controls	Borderline PD	*p*
Age	31.4 (8.4)	29.4 (7.9)	.229
Vocabulary test (verbal IQ)	31.6 (7.6)	30.3 (7.6)	.325
Psychopathological scores, mean (SD)			
Zanarini Interview	–	11.3 (6.5)	
BSL sum	41.9 (29.7)	198.1 (67.4)	<.001
FDS total	2.6 (2.9)	26.4 (21.1)	<.001
BDI	3.9 (4.7)	27.2 (12.7)	<.001
CTQ	43.3 (10.8)	74.7 (20.5)	<.001
BSI	0.3 (0.33)	1.8 (0.86)	<.001
SIAS	19.5 (10.4)	38.2 (15.1)	<.001
STAI	36.0 (11.0)	62.5 (10.1)	<.001
STAXI	16.8 (5.5)	27.2 (8.1)	<.001
Axis I comorbidity, No. (%)			
Substance abuse	–	11 (20.4)	
Major depression (current)	–	20 (37.0)	
Major depression (lifetime)	–	35 (64.8)	
Dysthymia	–	12 (22.2)	
Anxiety disorder	–	23 (42.6)	
Obsessive-compulsive disorder	–	7 (13.0)	
Post-traumatic stress disorder	–	16 (29.6)	
Eating disorder	–	13 (24.1)	
Axis II comorbidity, No.(%)			
Paranoid PD	–	5 (9.3)	
Schizoid PD	–	1 (1.9)	
Obsessive-compulsive PD	–	3 (5.6)	
Avoidant PD	–	14 (25.9)	
Dependent PD	–	1 (1.9)	
Negativistic PD	–	1 (1.9)	
Depressive PD	–	6 (11.1)	

*PD* personality disorder, *BSL* borderline symptom list, *FDS* questionnaire for dissociative symptoms, *BDI* Beck depression inventory, *CTQ* childhood trauma questionnaire, *BSI* brief symptom inventory, *SIAS* social interaction anxiety scale, *STAI* state-trait anxiety inventory, *STAXI* state-trait anger expression inventory

Comorbidity rates in BPD patients are given in Table 1. As medication is very common in BPD, we did not exclude medicated patients except those who were taking benzodiazepines. In the present sample, 39% (*n* = 20) of the patients were free of psychopharmacological medication. About 47% (*n* = 27) of the patients were taking antidepressant medication and 18% (*n* = 9) atypical antipsychotics.

### Cognitive empathy

**Fig. 3:**
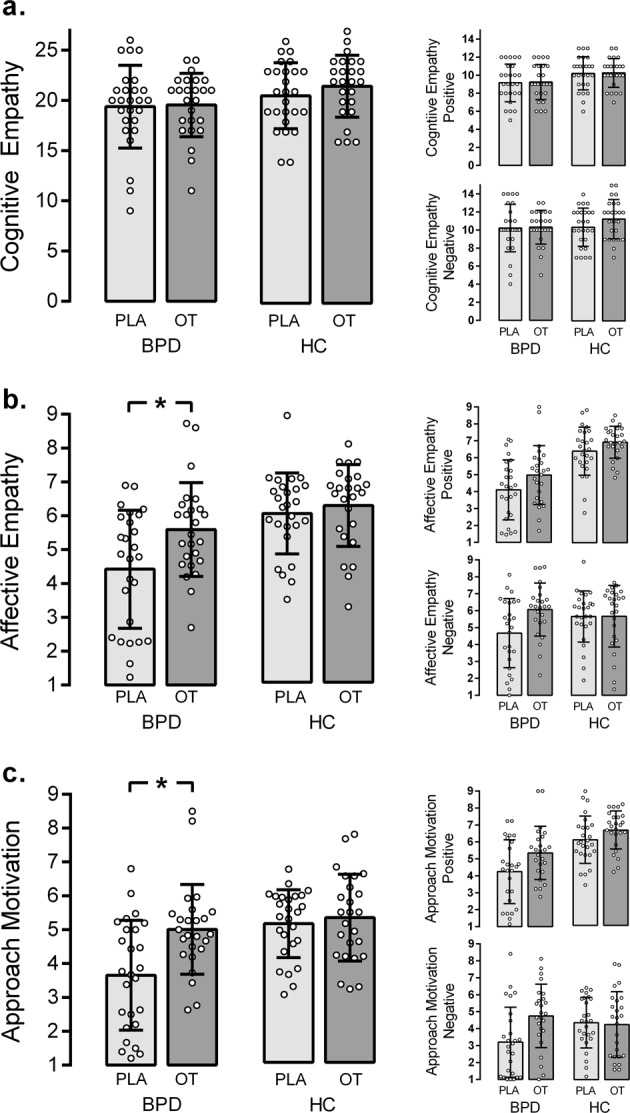
Main Results. Effects of oxytocin (OT) compared to placebo (PLA) on **a** cognitive and **b** affective empathy and **c** approach motivation in healthy controls (HC) and patients with borderline personality disorder (BPD). Results are given for total scores (left column) and for positive and negative stimuli separately (right column). Error bars represent SD. *p < .05.

The 3-way ANOVA on MET cognitive empathy (see Fig. 3a; Supplementary Table S1) revealed an overall significantly lower performance in patients with BPD compared to controls (main effect group: *F*_(1,98)_ = 5.63, *p* = .020, η^2^ = .054), but no main effect of OT (main effect drug: *F*_(1,98)_ = 0.68, *p* = .41) and no differential effect of OT in BPD patients and controls (group x drug interaction: *F*_(1,98)_ = 0.32, *p* = .57). In addition, the main effect of valence was significant (*F*_(1,98)_ = 13.7, *p* < .001; η^2^ = .123), while the valence x group interaction (*F*_(1,98)_ = 1.26, *p* = .265), valance x drug interaction (*F*_(1,98)_ = 0.90, *p* = .344) and valance x group x drug interaction (*F*_(1,98)_ = 0.89, *p* = .348) were not significant.

The ANOVAs for positive stimuli confirmed lower performance in BPD patients than controls (main effect group: *F*_(1,98)_ = 8.10, *p* = .005, η^2^ = .076), but again no effect of OT (main effect drug: *F*_(1,98)_ = 0.33, *p* = .856) and no drug-by-group interaction (*F*_(1,98)_ = 0.01, *p* = .961). For negative stimuli, none of the effects was significant (main effect group: *F*_(1,98)_ = 1.60, *p* = .208; main effect drug: *F*_(1,98)_ = 1.25, *p* = .266; interaction: *F*_(1,98)_ = 0.84, *p* = .362).

### Affective empathy

The 3-way ANOVA on MET affective empathy (see Fig. 3b; Supplementary Table S1) revealed significantly lower affective empathy in BPD patients (main effect group: *F*_(1,98)_ = 15.47, *p* < .001, η^2^ = .136), increased affective empathy following OT treatment (main effect drug. *F*_(1,98)_ = 6.43, *p* = .013, η^2^ = .062), but no group x drug interaction (*F*_(1,98)_ = 2.81, *p* = .097). In addition, the valence x group interaction was significant (*F*_(1,98)_ = 28.7, *p* < .001; η^2^ = .226), while the main effect of valence (*F*_(1,98)_ = 0.35, *p* = .553), valance x drug interaction (*F*_(1,98)_ = 0.01, *p* = 990.) and valence x group x drug interaction (*F*_(1,98)_ = 2.21, *p* = .140) did not reach significance.

Descriptive data suggest that affective empathy in BPD patients with OT reached the placebo level in healthy controls. Bonferroni-corrected *t*-tests for independent samples confirmed this impression: BPD patients with OT exhibited significantly higher affective empathy than BPD patients with placebo (*t*(49) = −2.643, *p* = .011, d = −.68). In addition, healthy controls with placebo and BPD patients with OT did not differ significantly (*t*(49) = −1.074, *p* = .288. d = .27).

For positive stimuli, BPD showed lower affective empathy than controls (main effect group: *F*_(1,98)_ = 51.31, *p* < .001, η^2^ = .344), OT treatment increased affective empathy in both groups (main effect drug: *F*_(1,98)_ = 5.52, *p* = .021, η^2^ = .053), whereas the group x drug interaction was not significant (*F*_(1,98)_ = 0.33, *p* = .568). For negative stimuli, the main effect of group was not significant (*F*_(1,98)_ = .67, *p* = .414). OT increased affective empathy in terms of a main effect (*F*_(1,98)_ = 4.16, *p* = .044, η^2^ = .041). In addition, the group x drug interaction was significant (*F*_(1,98)_ = 3.94, *p* = .049, η^2^ = .039) indicating that the effect of OT was more pronounced in BPD patients than controls.

### Approach motivation

For approach motivation (see Fig. 3c; Supplementary Table S1), the 3-way ANOVA revealed a significant main effect of group, with BPD patients showing less approach motivation than controls (*F*_(1,98)_ = 10.46, *p* = .002, η^2^ = .096). OT significantly increased approach motivation scores in terms of a main effect (*F*_(1,98)_ = 14.909, *p* = .004, η^2^ = .079). The significant group x drug interaction (*F*_(1,98)_ = 8.87, *p* = .004, η^2^ = .049) indicates that OT-induced increase in approach motivation was more pronounced in BPD patients than healthy controls. Notably, approach motivation scores in BPD patients with OT and healthy controls with placebo did not differ. Pair-wise comparisons confirmed that BPD patients with OT exhibited significantly more approach motivation than BPD patients with placebo (*t*(49) = −3.255, *p* = .002, d = −.91) and that BPD patients with OT reached the level of healthy controls with placebo (*t*(49) = −.261, *p* = .795. d = −.08). In addition, main effect of valance (*F*_(1,98)_ = 43.2, *p* < .001; η^2^ = .306) and valence x group interaction were significant (*F*_(1,98)_ = 7.78, *p* *=* .006; η^2^ = .074), while the valence x drug (*F*_(1,98)_ = 0.08, *p* *=* .776) and valence x group x drug interaction were not significant (*F*_(1,98)_ = 1.68, *p* *=* .198).

For approach motivation towards positive stimuli, the ANOVA indicated lower overall approach motivation in BPD patients (main effect group: *F*_(1, 98)_ = 26.78, *p* < .001, η^2^ = .212) and a significant enhancing overall effect of OT (main effect drug: *F*_(1, 98)_ = 7.81, *p* = .006, η^2^ = .074), whereas the group x drug interaction was not significant (*F*_(1, 98)_ = 0.77, *p* = .382). For negative stimuli, BPD patients did not differ significantly from controls (main effect group: *F*_(1, 98)_ = 0.81, *p* = .372, η^2^ = .008), and OT had no general impact in terms of a main effect (*F*_(1, 98)_ = 3.83, *p* = .053). However, the group x drug interaction was significant, indicating that the effect of OT was more pronounced in BPD patients than controls (*F*_(1, 98)_ = 5.12, *p* = .026, η^2^ = .050).

The effects within the BPD group were not moderated by symptom severity as ANCOVAs with BSL (Cognitive Empathy: *F*_(1,48)_ = 0.805, *p* = .374, η^2^ = .017; Affective Empathy: *F*_(1,48)_ = 0.106, *p* = .746, η^2^ = .002; Approach Motivation: *F*_(1,48)_ = 0.025, *p* = .875, η^2^ = .001) and CTQ (Cognitive Empathy: *F*_(1,48)_ = 0.07, *p* = .792, η^2^ = .001; Affective Empathy: *F*_(1,48)_ = 2.729, *p* = .105, η^2^ = .054; Approach Motivation: *F*_(1,48)_ = 3.448, *p* = .069, η^2^ = .067) scores as covariates revealed no significant results.

### Non-specific oxytocin effects

To control for non-specific effects of OT over the course of the experiment, we tested for OT effects on self-reported mood, anxiety, anger, and distress. Values for mood increased over time, whereas anxiety and anger values decreased over time and distress did not change over time. We observed no significant interaction (time x group x drug) in any of the measures. In sum, OT had no non-specific effect on mood, anxiety, anger and distress (see supplemental results for a detailed description; descriptive data is given in Supplementary Table S2).

## Discussion

In the present study, patients with BPD showed reduced cognitive and affective empathy, and less approach behavior motivation than a healthy control group, especially for positive social stimuli. A single-dose of OT given intranasally did not significantly influence cognitive empathy, but did significantly enhance affective empathy and approach behavior motivation in BPD patients and healthy controls. Patients with BPD who received OT attained a level of affective empathy and approach motivation similar to healthy controls with placebo. For positive social stimuli, both groups exhibited comparable benefits from OT administration. For negative stimuli, BPD patients showed remarkable improvements under OT, whereas healthy controls revealed no OT-induced alterations.

Our findings of impaired cognitive and affective empathy in BPD are in line with previous studies using the same experimental task^12^ as well as studies applying different measures to assess empathy in BPD^14,41,42^. Our findings support the hypothesis that apart from heightened sensitivity for social cues in BPD, patients exhibit deficits in integrating complex social information, which may contribute to social impairments and interpersonal difficulties^14,43^. Importantly, in analyzing our data for positive and negative stimuli separately, we show that empathy and approach motivation alterations in BPD are mainly driven by impaired responding to positive stimuli, while there is no such difference for negative stimuli. It appears that BPD patients more easily empathize with people in aversive situations or in distress, while it is difficult for them to be empathic with people in positive social situations or people experiencing positive emotions. This pattern is plausible, as negative emotions and situations are much more familiar to patients with BPD and thus negative emotions might be more easily accessible for BPD patients^44^.

A recent meta-analysis showed that OT enhances facial emotion recognition, which conceptually overlaps with the cognitive facet of empathy as measured with the MET^25^. In contrast, we found no enhancing effect of OT on cognitive empathy in the present study. We assume that this might be due to differences in the experimental task or stimuli presented, as most previous studies used emotional faces or pictures of the eye region. In the present study, we presented complex scenes of individuals in emotionally-laden situations, challenging the participants’ ability to integrate context information with facial expressions and perspective taking to infer complex affective mental states rather than recognize basic emotions. On the other hand, our results are in line with studies that tested the effects of OT in healthy samples using the MET and reported no substance effect on cognitive empathy but an enhancing effect on affective empathy^27,45^. Further evidence arises from studies using empathy to others’ pain as a paradigm to assess OT’s effects on affective empathy e.g., ref. ^46^.

Aberrant approach behavior motivation in the BPD group concurs with clinical experiences in BPD, emphasizing rapid alterations between over-involvement and withdrawal as one of the core symptoms of the disorder^3^ and findings from attachment research^47^. Extending the standard MET, the present study provides the first experimental evidence for reduced approach motivation in BPD compared to healthy controls. Notably, we found that OT enhanced approach motivation in our sample using this experimental paradigm to measure approach motivation. It should be noted that affective empathy and approach motivation correlated closely. Thus, affective empathy might be considered the socio-affective prerequisite of the desire to approach others. It is also possible that the reverse is true or that both affective empathy and approach motivation are related to other variables and that OT increased liking or general interest in the other person. There is strong evidence in animal studies of the approach enhancing effects of OT^48^ and these have been also repeatedly reported in humans^15^. Thus, OT’s beneficial effects in terms of enhancing approach motivation in BPD to a normal level might be a promising starting point for OT-enhanced behavioral interventions in situations of dysfunctional avoidance. However, it is important to keep in mind that in certain other situations, increased approach behavior might also be maladaptive for BPD patients, requiring careful therapeutic supervision. Moreover, since BPD is also known for maladaptive approach behavior in social interaction (e.g., excessive anger expression), future studies will need to investigate adaptive versus maladaptive approach situations further. As the level of approach in BPD under OT was similar to healthy controls in our study, our findings do not reflect a maladaptive ‘overapproaching’ but rather a normalized approach. Future clinical trials are needed to provide direct evidence for the usefulness of exogenous OT in BPD and other disorders characterized by altered social approach, e.g., autism spectrum conditions^49–51^ and social anxiety disorder^52^.

Although OT increased empathy and motivational approach to positive stimuli in both BPD patients and healthy controls to the same extent, the effect was more pronounced in BPD patients for negative stimuli. Previous studies have shown that BPD patients exhibit higher trait anxiety and enhanced limbic activity in response to threat-related social stimuli^31^. In addition, a previous functional imaging study provided evidence for reduced amygdala reactivity in female BPD patients after OT treatment^31^. Thus, in the present study, the OT increase in empathy and approach motivation to negative stimuli might be attributable to OT-induced reduction of amygdala-related arousal. Given that earlier studies as well as this study’s results suggest that BPD patients have difficulty processing positive social cues, stimulus valence needs further investigation in the future.

This study has some limitations. First of all, only female participants were investigated. As the effects of exogenous OT might be different for women and men^53^, the present results might not apply to male patients. Furthermore, both empathy and approach motivation were assessed using self-reported ratings, although we chose a stimulus-associated approach. Future studies are needed to confirm the present results using situations explicitly triggering empathic and approach-related behavior. In addition, as we included patients taking psychoactive medication targeting serotonergic and dopaminergic neurotransmission, we cannot exclude interactions between OT and medication in these patients. Finally, since we had no clinical control group, our findings’ specific validity will need to be investigated in future studies by comparing different clinical groups or using a dimensional approach with participants varying in their social avoidance and empathy.

## Conclusion

The present study demonstrates for the first time that a single dose of OT given intranasally improves affective empathy and approach motivation in women with BPD. Our results may have clinical implications, as they suggest that socio-affective deficits in BPD might be specifically targeted via an intervention in the OT system. These results could provide the starting point for designing controlled clinical trials, focusing on treatment efficiency using OT as an add-on treatment to cognitive behavioral psychotherapy in BPD, while taking practical issues into account such as the route of delivery of the peptide^54^, as well as timing and dosage^55^.

## Supplementary information

Supplementary information
