# Supplementary material for: Effects of intranasal oxytocin administration on empathy and approach motivation in women with borderline personality disorder: a randomized controlled trial
Source: Transl Psychiatry. 2019 Dec 4;9:328. doi: 10.1038/s41398-019-0658-4 (PMC6892895; doi:10.1038/s41398-019-0658-4)
Supplement: Supplementary file 1 — Supplementary information [file 41398_2019_658_MOESM1_ESM.pdf]

Supplementary Information

Effects of intranasal oxytocin administration on empathy and approach motivation in women with borderline personality disorder – a randomized trial

Gregor Domes[1,2]*, PhD; Nicole Ower[1]*, PhD; Bernadette von Dawans[1,2], PhD; Franny B. Spengler[1], PhD; Isabel Dziobek[3], PhD; Martin Bohus[4], MD; Swantje Matthies[5], MD; Alexandra Philipsen[6], MD; PhD; Markus Heinrichs[1,7], PhD

* Equal contribution

[1] Department of Psychology, Laboratory for Biological and Personality Psychology, University of Freiburg, Freiburg, Germany

[2] Department of Biological and Clinical Psychology, University of Trier, Germany

[3] Department of Psychology, Social Cognition, Humboldt University Berlin, Germany

[4] Department of Psychosomatic Medicine and Psychotherapy, Central Institute of Mental Health, University of Heidelberg, Mannheim, Germany

[5] Department of Psychiatry and Psychotherapy, Medical Center, University of Freiburg, Germany

[6] Department of Psychiatry and Psychotherapy, University Hospital Bonn, Germany

[7] Freiburg Brain Imaging Center, University Medical Center, University of Freiburg, Germany

Supplemental Methods

Sample size estimation

Total sample size was estimated based on previously reported medium effect sizes of oxytocin on socio-affective cognition. Based on a medium effect size of f=.30 (equivalent η_{part}^2=.08), alpha=.05 and a power of 1-beta=.80 a priori sample size calculation with GPower[1] revealed an appropriate size of at least N=92.

Questionnaires

All participants completed the following psychopathological measures: the Borderline Symptom List [2], the German adaption of the Dissociative Experience Scale [3], the Brief Symptom Inventory [4], the

Beck Depression Inventory [5], the Childhood Trauma Questionnaire [6], the Social Interaction and Anxiety Scale [7], the State-Trait Anxiety Inventory [8] and the State-Trait-Anger Expression Inventory [9].

To control for non-specific drug effects, we collected the following state measures: mood, using the Multimodal Mood Questionnaire [10], anxiety using the State-Trait-Anxiety-Inventory [8], anger using the State-Trait-Anger-Inventory [9]

Supplemental Results

Table S1. Descriptive data on cognitive and affective empathy, and approach motivation in patients with Borderline PD and healthy controls treated with oxytocin (OT) or placebo (PLA).

	Healthy controls		Borderline PD	
	PLA	OT	PLA	OT
Cognitive empathy, mean (SD)				
All stimuli	20.62 (3.28)	21.56 (3.07)	19.38 (4.12)	19.56 (3.16)
Positive stimuli	10.23 (1.84)	10.28 (1.59)	9.15 (2.09)	9.24 (1.94)
Negative stimuli	10.38 (2.12)	11.28 (2.17)	10.23 (2.64)	10.32 (1.86)
Affective empathy, mean (SD)				
All stimuli	5.98 (1.19)	6.22 (1.21)	4.43 (1.74)	5.59 (1.67)
Positive stimuli	6.42 (1.42)	6.95 (0.95)	4.11 (1.77)	4.98 (1.74)
Negative stimuli	5.64 (1.51)	5.66 (1.81)	4.67 (2.04)	6.06 (1.56)
Approach motivation, mean (SD)				
All stimuli	5.10 (1.00)	5.27 (1.28)	3.66 (1.62)	5.01 (1.33)
Positive stimuli	6.05 (1.40)	6.63 (1.12)	4.24 (1.88)	5.35 (1.57)
Negative stimuli	4.36 (1.49)	4.25 (1.93)	3.20 (2.07)	4.75 (1.87)

Non-specific drug effects

To control for non-specific drug effects, we collected the following measures: mood, using the Multimodal Mood Questionnaire (MBDF, Steyer, Schwenkmezger, Notz, & Eid, 1997), anxiety using the State-Trait-Anxiety-Inventory (STAI; Laux, Glanzmann, Schaffner, & Spielberger, 1981), anger using the State-Trait-Anger-Inventory (STAXI; Schwenkmezger, Hodapp, & Spielberger, 1992) and distress with a visual analog scale (VAS) at three times during the time-course of the experiment: before drug application (t_1), shortly before the experiment (t_2) and directly after the experiment (t_3). Descriptive statistics can be found in table S2.

Table S2. Scores for mood, anxiety, anger and distress (MDBF and VAS scores) in patients with borderline personality disorder (BPD) and healthy controls (HC) treated with oxytocin (OT) or placebo (PLA).

	HC		BPD	
	PLA (n=	OT	PLA	OT
Pre-application (t_1) mean (SD)				
Mood (MDBF 5-20)	15.42 (1.65)	15.60 (2.02)	8.96 (3.49)	9.72 (3.70)
Anxiety (STAI-State 20-80)	28.69 (11.12)	34.28 (7.28)	54.69 (14.12)	52.12 (14.91)
Anger (STAXI-State 4-40)	9.50 (2.69)	10.24 (0.66)	14.35 (5.50)	14.40 (6.39)
Distress (VAS 0-500)	59.77 (54.04)	51.04 (34.55)	210.54 (125.57)	213.28 (143.13)
Post-application (t_2), mean (SD)				
Mood (MDBF 5-20)	15.12 (2.01)	15.40 (2.25)	10.15 (3.70)	9.80 (3.61)
Anxiety (STAI-State 20-80)	32.38 (6.39)	30.08 (5.56)	48.15 (11.08)	48.84 (11.00)
Anger (STAXI-State 4-40)	10.38 (0.98)	10.16 (0.55)	13.00 (4.64)	12.36 (4.37)
Distress (VAS 0-500)	52.92 (73.92)	27.80 (29.28)	150.23 (105.12)	174.00 (138.40)
Post-experiment (t_3), mean (SD)				
Mood (MDBF 5-20)	15.96 (1.43)	16.48 (1.58)	11.15 (4.16)	11.24 (2.89)
Anxiety (STAI-State 20-80)	30.88 (4.57)	28.72 (5.10)	47.50 (11.27)	46.72 (8.95)
Anger (STAXI-State 4-40)	10.08 (0.39)	10.12 (0.33)	12.58 (4.55)	11.92 (4.31)
Distress (VAS 0-500)	42.85 (53.10)	24.28 (32.03)	161.15 (118.25)	169.12 (136.81)

We first conducted a two-way ANOVA to control for differences at baseline level. The factor group showed a significant main effect for all variables: mood ($F_{(1, 98)}$ = 118.9, p < .001, η^2 = .548), anxiety ($F_{(1, 99)}$ = 81.8, p < .001, η^2 = .455), anger ($F_{(1, 99)}$ = 26.3, p < .001, η^2 = .212) and distress ($F_{(1, 99)}$ = 61.8, p < .001, η^2 = .387). BPD patients revealed significantly lower values for mood and significantly higher values for anxiety, anger, and distress.

To further analyze changes in these measures along the time-course, we conducted a three-way ANOVA with repeated measures (time). We identified significant main effects of time on the variables mood ($F_{(2, 196)}$ = 16.4, p < .001, η^2 = .143), anxiety ($F_{(1.4, 141.5)}$ = 6.65, p = .005, η^2 = .064) , and distress ($F_{(1.6, 161.1)}$ = 7.42, p = .001, η^2 = .070). The time effect for anger was not significant ($F_{(1.2, 119.7)}$ = 3.48, p = .056, η^2 = .034). Values for mood increased over time, whereas anxiety and stress decreased over time.

We observed no main effect of drug and no interaction of drug with either group or time. Thus, OT exerted no nonspecific effects on the measures, and the changes over the time course did not significantly differ between the BPD and HC group or between the OT and placebo condition.

Supplemental References

1 Faul F, Erdfelder E, Buchner A, Lang A-G. Statistical power analyses using G*Power 3.1: Tests for correlation and regression analyses. *Behavior Research Methods* 2009; **41**: 1149–1160.

2 Bohus M *et al.* Psychometric properties of the Borderline Symptom List (BSL). *Psychopathology* 2007; **40**: 126–32.

3 Stuhldreher N *et al.* The costs of social anxiety disorder: the role of symptom severity and comorbidities. *J Affect Disord* 2014; **165**: 87–94.

4 Franke GH. *Brief Symptom Inventory*. Beltz: Göttingen, 2000.

5 Hautzinger M, Worall H, Keller F. *Beck Depressionsinventar (BDI)*. Huber: Bern, 1994.

6 Bernstein DP *et al.* Initial reliability and validity of a new retrospective measure of child abuse and neglect. *Am J Psychiatry* 1994; **151**: 1132–6.

7 Stangier U, Heidenreich T, Berardi A, Golbs U, Hoyer J. Die Erfassung sozialer Phobie durch die Social Interaction Anxiety Scale (SIAS) und die Social Phobia Scale (SPS). *Zeitschrift für Klinische Psychologie und Psychotherapie* 1999; **28**: 28–36.

8 Laux L, Glanzmann P, Schaffner P, Spielberger CD. Das State-Trait-Angstinventar (STAI) [The state-trait anxiety inventory]. *Göttingen, Bern Hogrefe* 1981.

9 Schwenkmezger P, Hodapp V, Spielberger C. *Das State-Trait-Ärgerausdrucks-Inventar (STAXI) [Teh state-trait anger expression inventory]*. Huber, 1992.

10 Steyer R, Schwenkmezger P, Notz P, Eid M. Der mehrdimensionale Befindlichkeitsfragebogen (MDBF). Handanweisung [The multidimensional affect rating scale (MDBF). Manual]. *Göttingen, Germany: Hogrefe* 1997.
